# The Matrisome Is Associated with Metabolic Reprograming in Stem-like Phenotypes of Gastric Cancer

**DOI:** 10.3390/cancers14061438

**Published:** 2022-03-10

**Authors:** Ji-Yong Sung, Jae-Ho Cheong

**Affiliations:** 1 Department of Laboratory Medicine, Yonsei University College of Medicine, Seoul 03722, Korea; 2 Department of Surgery, Yonsei University College of Medicine, Seoul 03722, Korea; 3 Yonsei Biomedical Research Institute, Yonsei University College of Medicine, Seoul 03722, Korea; 4 Department of Biochemistry & Molecular Biology, Yonsei University College of Medicine, Seoul 03722, Korea

**Keywords:** matrisome, epithelial-mesenchymal transition, stem-like gastric cancer, glycosaminoglycan biosynthesis-chondroitin sulfate, extracellular matrix

## Abstract

**Simple Summary:**

Our results suggested a correlation between the metabolic reprogramming associated with the high-matrisome group and stem-like phenotype in gastric cancer. Carbohydrate sulfotransferase 7 was found to be associated with the signaling transduction of overexpressed oncogenes and tumor suppressor genes in the high-matrisome group. The high expression of glycosaminoglycan biosynthesis-chondroitin sulfate metabolic pathway genes was associated with poor prognosis.

**Abstract:**

The extracellular matrix (ECM) is an important regulator of all cellular functions, and the matrisome represents a major component of the tumor microenvironment. The matrisome is an essential component comprising genes encoding ECM glycoproteins, collagens, and proteoglycans; however, its role in cancer progression and the development of stem-like molecular subtypes in gastric cancer is unknown. We analyzed gastric cancer data from five molecular subtypes (*n* = 497) and found that metabolic reprograming differs based on the state of the matrisome. Approximately 95% of stem-like cancer type samples of gastric cancer were in the high-matrisome category, and energy metabolism was considerably increased in the high-matrisome group. Particularly, high glycosaminoglycan biosynthesis-chondroitin sulfate metabolic reprograming was associated with an unfavorable prognosis. Glycosaminoglycan biosynthesis-chondroitin sulfate metabolic reprograming may occur according to the matrisome status and contribute to the development of stem-like phenotypes. Our analysis suggests the possibility of precision medicine for anticancer therapies.

## 1. Introduction

The extracellular matrix (ECM) is most commonly defined as the noncellular component of tissues [1]. ECM is an essential element for cellular homeostasis, and it regulates the tumor microenvironment and influences cancer progression and development [2]. The matrisome is mainly composed of genes encoding ECM glycoproteins, collagens, and proteoglycans [3]. Recently, studies on the matrisome and its immunosuppressive function have been published [4]; however, the matrisome and metabolic reprograming associated with gastric cancer have not yet been elucidated. The tumor microenvironment [5] exists between multiple cells competing for nutrition with the tumor cells via signal transduction.

Metabolic reprograming affects the function of immune cells and ECM that regulates the tumor microenvironment [2]. The prognosis of patients with specific cancers differs depending on the status of the matrisome [6]. The stem-like phenotype, which is associated with the worst prognosis for gastric cancer [7], is an aggressive tumor type that shows drug resistance and characteristics of cancer stem cells that have undergone autophagy.

Changes in the ECM modulate a number of intracellular signaling pathways [8]. These alterations in the tumor microenvironment regulate cell adhesion, differentiation, invasion, metabolism, migration, proliferation, survival, chemosensitivity, cytoskeletal dynamics, epithelial-to-mesenchymal transition (EMT), matrix synthesis, remodeling enzyme secretion, and stem-cell-like behavior [2]. Therefore, it is necessary to understand the molecular mechanisms associated with a high-matrisome-type gastric cancer and to determine vulnerable stem-like subtypes as targets for treatment. Here, using the transcriptome data of four gastric cancer cohorts (Gene Expression Omnibus series (GSE) 15459, GSE62254, The Cancer Genome Atlas (TCGA) stomach adenocarcinoma (STAD), and our cohort Y497), we compared the high-matrisome group and low-matrisome group based on a matrisome signature score. Our approach represents a starting point for understanding refractory cancer with respect to precision medicine and for developing new drugs.

## 2. Results

### 2.1. Glycosaminoglycan Biosynthesis-Chondroitin Sulfate Metabolic Reprograming Is Correlated with High-Matrisome Scores

**Figure 1:**
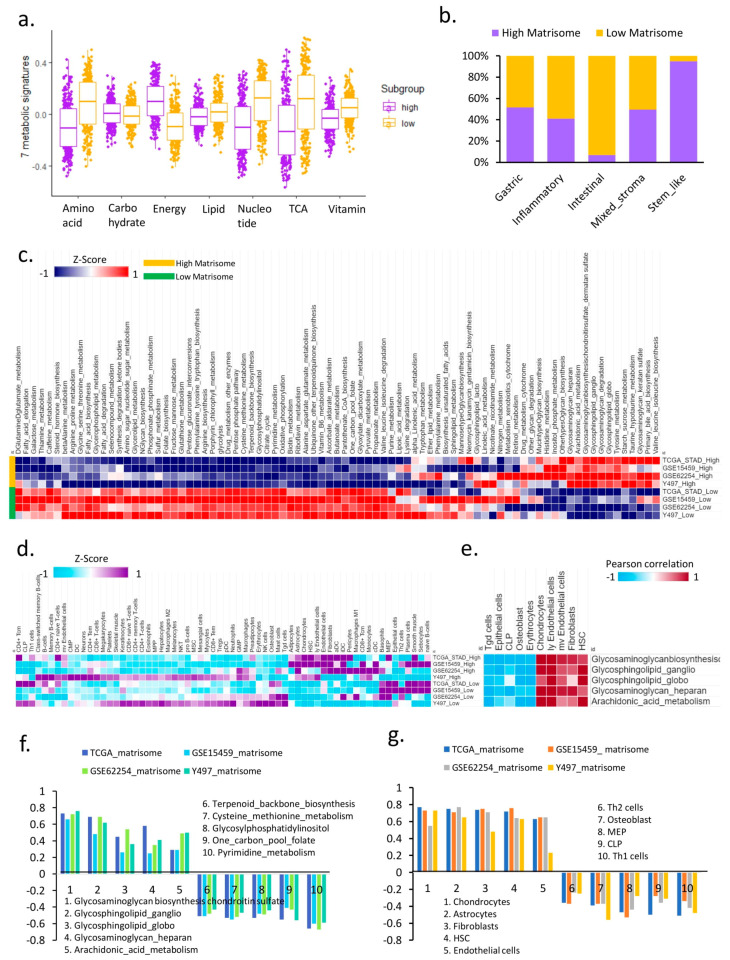
The landscape of metabolic reprograming between high-matrisome and low-matrisome groups. (**a**) Box plot showing seven metabolic signatures between high-matrisome and low-matrisome in the Y497 cohort. (**b**) Bar graph showing matrisome state across five molecular subtypes. (**c**) Heat map showing activity of 83 metabolic pathways in high-matrisome and low-matrisome groups. (**d**) Heat map showing 22 cell types in high-matrisome and low-matrisome groups. (**e**) Heat map showing Pearson correlation between top enriched metabolic pathways and top ranked cell types. (**f**) Bar graph of Pearson correlation between matrisome values and metabolic pathway gene expression levels. (**g**) Bar graph of Pearson correlation between matrisome values enriched in various cell types.

The tumor microenvironment undergoes metabolic reprograming [9], and the ECM influences the tumor microenvironment as a regulator of cancer progression [10]. However, the involvement of the ECM in metabolic reprograming in the tumor microenvironment has not been investigated extensively. We defined the gastric cancer data of five molecular subtypes as samples showing a higher-than-average value according to the matrisome status as a high-matrisome group and samples showing a lower-than-average matrisome value as a low-matrisome group, and compared seven metabolic signatures (lipid, carbohydrate, tricarboxylic acid cycle (TCA), energy, nucleotide, vitamin, and amino acid) between the two groups. The expression of genes for energy metabolism (*p* < 2.2 × 10^−16^) and four other metabolic pathways (amino acid: 2.2 × 10^−16^ lipid: *p* = 0.00017, nucleotide: *p* < 2.2 × 10^−16^, TCA: *p* < 2.2 × 10^−16^, and vitamin in the low-matrisome group) was significantly increased in the high-matrisome group, while there was no difference in carbohydrate metabolism (*p* = 0.1) (Figure 1a). In particular, among the five molecular subtypes (gastric, inflammatory, intestinal, mixed stroma, stem-like), 95% of samples among the stem-like subtypes were in the high-matrisome category (Figure 1b).

To further analyze metabolic reprograming, we classified the four gastric cancer cohorts according to their matrisome status and analyzed 83 Kyoto Encyclopedia of Genes and Genome (KEGG) metabolic pathways. In all four cohorts, the enrichment of high glycosaminoglycan biosynthesis-chondroitin sulfate metabolic activity was significantly greater in the high-matrisome group than that in the low-matrisome group (FDR < 0.001) (Figure 1c). We compared the differences according to the matrisome status in 64 cell types using xCell [11] to analyze the landscape of metabolite programing and the tumor immune microenvironment (Figure 1d). The correlation analysis of each cell type, together with metabolic pathways, confirmed that high expression of genes in five activated metabolic pathways (glycosaminoglycan biosynthesis-chondroitin sulfate, glycosphingolipid biosynthesis-ganglio series, glycosphingolipid biosynthesis-globo and isoglobo series, glycosaminoglycan biosynthesis-heparan sulfate, and arachidonic acid metabolism) in the high-matrisome group was enriched in chondrocytes, lymphatic endothelial cells, microvascular endothelial cells, fibroblasts, and hematopoietic stem cells (HSCs), whereas gamma-delta T cells, epithelial cells, common lymphoid progenitors (CLPs), and osteoblasts were negatively correlated with these five metabolic pathways (Figure 1e).

We analyzed the correlation between high matrisome and metabolism, and the strongest positive correlations between gene expression in metabolic pathways with high matrisome were as follows: glycosaminoglycan biosynthesis-chondroitin sulfate (R = 0.66~0.76), glycosphingolipid biosynthesis-ganglio series (R = 0.48~0.69), glycosphingolipid biosynthesis-globo and isoglobo series (R = 0.36~0.54), glycosaminoglycan biosynthesis-heparan sulfate (R = 0.25~0.58), and arachidonic acid metabolism (R = 0.29~0.5). The five metabolic pathways were negatively correlated with the matrisome signature (Figure 1f). Based on cell type, the top five cell types with the highest matrisome values were chondrocytes, astrocytes, fibroblasts, HSCs, and endothelial cells, and the lowest five cell types were Th2 cells, osteoblasts, megakaryocyte–erythroid progenitors, CLPs, and Th1 cells (Figure 1g). Dermatan sulfate proteoglycan and glycosaminoglycan synthesis is induced in fibroblasts by ECM [12]. Overall, our results showed a correlation between the metabolic reprograming associated with the high-matrisome group and the stem-like phenotype in gastric cancer.

### 2.2. The ECM May Modulate the Hallmarks of Cancer

**Figure 2:**
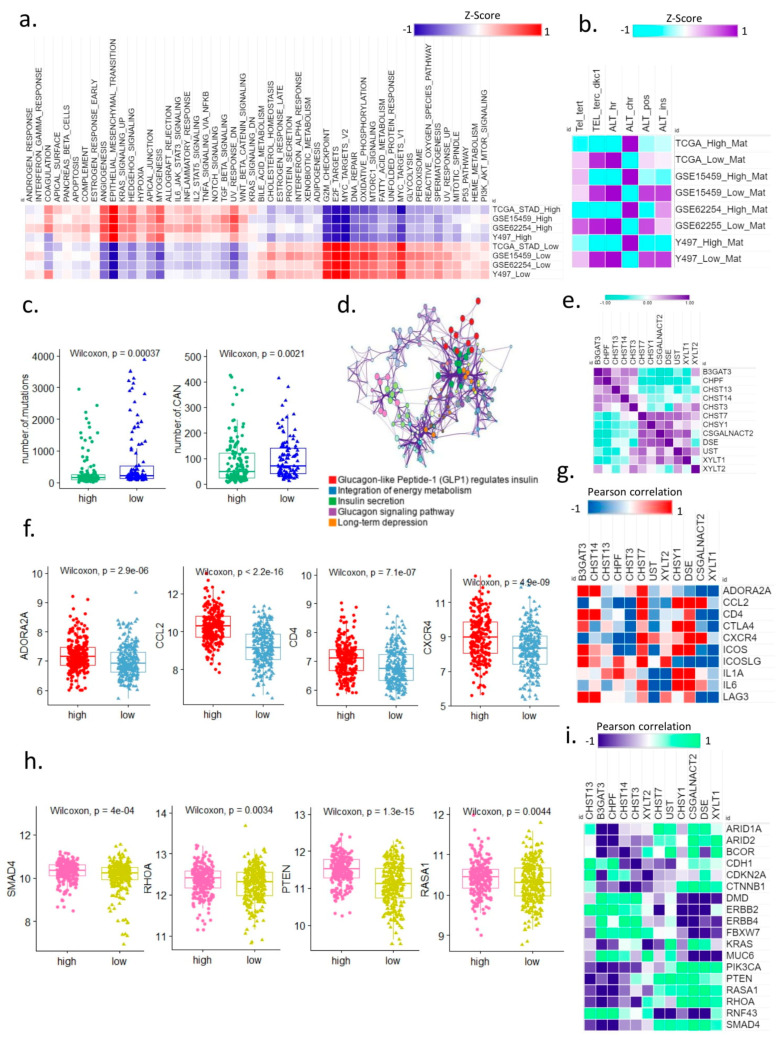
The extracellular matrix modulates the hallmarks of cancer. (**a**) Heat map of 50 cancer hallmarks in the high-matrisome group and low-matrisome group in 4 gastric cancer cohorts. (**b**) Heat map of telomere maintenance mechanism in the high- and low-matrisome groups in 4 gastric cohorts. (**c**) Boxplot of mutation burden in high- and low-matrisome groups in STAD (left); boxplot of copy alternation number in high- and low-matrisome groups in the STAD cohort (right). (**d**) Network of biological pathways of integrated energy metabolism with elevated gene expressions enriched in high-matrisome samples (4 cohorts). (**e**) Heat map of similarity for Pearson correlation between glucagon-like peptide 1-regulated insulin secretion genes and glycosaminoglycan biosynthesis-chondroitin sulfate genes in cohort Y497. (**f**) Boxplot of overexpressed immune checkpoint target genes in high-matrisome samples in cohort Y497. (**g**) Heat map of Pearson correlation between highly expressed immune checkpoint target genes and highly expressed glycosaminoglycan biosynthesis-chondroitin sulfate genes in cohort Y497. (**h**) Boxplot of overexpressed oncogenes/tumor suppressor genes in high- and low-matrisome groups. (**i**) Heat map of Pearson correlation between highly expressed glycosaminoglycan biosynthesis-chondroitin sulfate genes and highly expressed oncogenes/tumor suppressor genes in cohort Y497. STAD, The Cancer Genome Atlas stomach adenocarcinoma.

We distinguished 50 cancer hallmarks according to their matrisome status to analyze the relevance of the matrisome to the cancer hallmarks (Figure 2a). Expression of genes for EMT and angiogenesis were high in the high-matrisome samples, and those of G2M checkpoint, E2F targets, and MYC targets were high in low-matrisome samples (Figure 2a). The high-matrisome group was associated with EMT cancer, and we found that the high-matrisome samples were enriched in alternative lengthening of telomere (ALT)-like cancer type, in which telomerase was not released in the telomere maintenance mechanism (TMM) analysis, and the activity of ALT chromatin decompaction was high [13]. ALT is an aggressive type that appears mainly with the stem-like phenotype of gastric cancer. In the four gastric cancer cohorts, the high-matrisome samples were enriched for the TMM type with no or low telomerase (Figure 2b). In addition, the matrisome can suppress the immune response and can be used as a cancer hallmark to predict the outcome of immunotherapy treatment. In particular, it is known that cancer immunotherapy is more effective when the tumor mutation burden (TMB) is high. The TMB was significantly (*p* = 0.00037) higher in the low-matrisome samples than in the high-matrisome samples, and the copy number alteration was also significantly (*p* = 0.0021) higher in the low-matrisome group (Figure 2c) than in the high-matrisome group.

We analyzed the expression of 10 immune checkpoint target genes in the two matrisome groups, and the expression of adenosine A2A receptor (*ADORA2A*), C–C motif chemokine ligand 2 (*CCL2*), cluster of differentiation 4 (*CD4*), and C-X-C chemokine receptor type 4 (*CXCR4*) was significantly upregulated in the high-matrisome group (Figure 2f). CCL2 signaling is critical for macrophage-mediated endocytosis of collagen and fibrin [14]. CXCR4 and integrin signaling play a role in mediating adhesion and chemoresistance [15]. ADORA2A activation promotes the transcriptional induction of glycolytic enzymes via extracellular signal-regulated kinase- and protein kinase B (Akt)-dependent translational activation of hypoxia-inducible factor 1α protein [16]. T lymphocyte adhesion to the fibronectin and laminin components of the extracellular matrix is regulated by the CD4 molecule [17].

In our cohort, correlation analysis of glycosaminoglycan biosynthesis-chondroitin sulfate metabolic pathway genes and 10 immune checkpoint target genes showed that the high expression of carbohydrate sulfotransferase 7 (*CHST7*) was strongly correlated with that of these 10 immune checkpoint blockade target genes (Figure 2g).

In oncogene and tumor suppressor gene analysis, mothers against decapentaplegic homolog 4, alkaline phosphatase, phosphatase and tensin homolog (*PTEN)*, and RAS p21 protein activator 1 were significantly upregulated in the high-matrisome group. Matrix stiffness induces the expression of microRNAs that downregulate the tumor suppressor PTEN, thereby enhancing phosphoinositide 3-kinase/Akt activity to promote cell growth and survival [18]. High-matrisome samples are predicted to have a loosely packed structure, as in EMT cancer [19]. ECM provides mechanical support to induce cancer stemness [20]. Modulation of Ras homolog family member A activity in vascular smooth muscle cells increases the activation of stress fibers [21].

Glycosaminoglycan biosynthesis-chondroitin sulfate metabolic pathway genes were positively or negatively correlated with oncogenes and tumor suppressor genes of gastric cancer, respectively, whereas high expression of *CHST7* was positively correlated with that of four significantly upregulated genes (Figure 2i).

In summary, the high-matrisome samples were enriched in aggressive EMT-like [22] and ALT-like phenotype tumors with low TMB and low copy number alteration and resistance to immunotherapy. High levels of *CHST7* were found to be associated with signal transduction of overexpressed oncogenes and tumor suppressor genes in the high-matrisome group.

### 2.3. The High-Matrisome Group Is Associated with Poor Prognosis in Gastric Cancer

**Figure 3:**
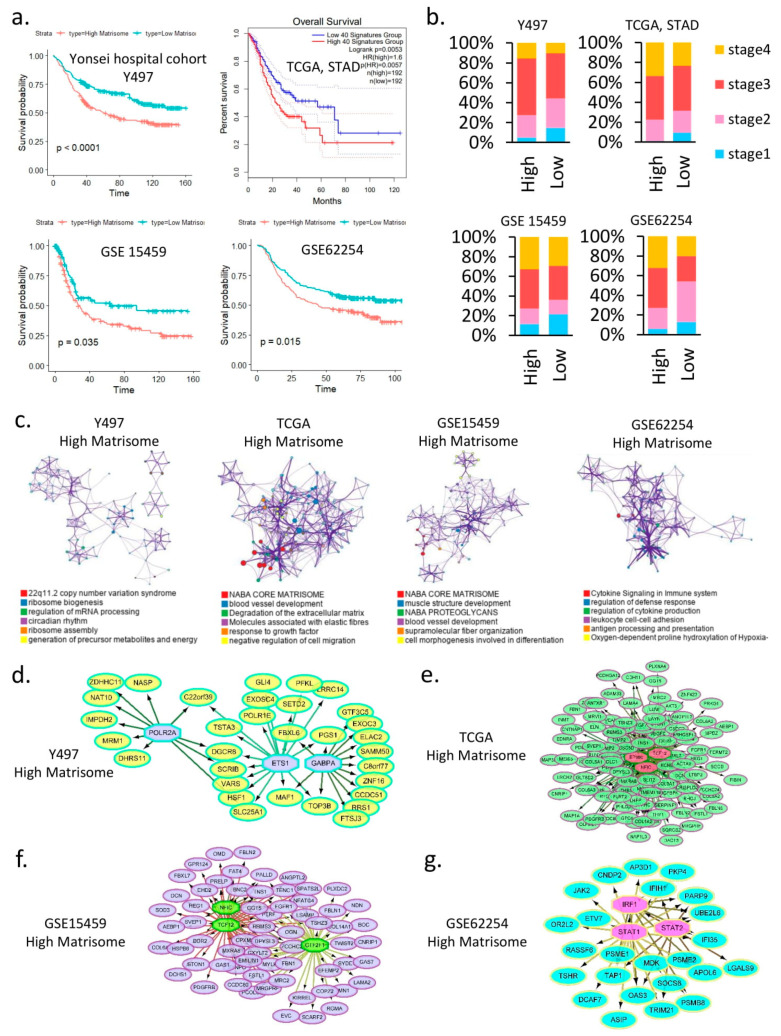
High-matrisome type is associated with poor prognosis in gastric cancer. (**a**) Kaplan–Meier plots show the overall survival rates for the high- and low-matrisome groups. The *p*-values were analyzed using the log-rank test and adjusted by Bonferroni correction. (**b**) Bar graph showing the tumor stages for the high- and low-matrisome groups. (**c**) Network of gene ontology analysis of high-matrisome groups of 4 cohorts (FDR < 0.001). (**d**) Network of transcription factor target genes and transcription factors in the high-matrisome group in cohort Y497. (**e**) Network of transcription factor target genes and transcription factors in the high-matrisome group in STAD cohort. (**f**) Network of transcription factor target genes and transcription factors in the high-matrisome group in cohort GSE15459. (**g**) Network of transcription factor target genes and transcription factors in the high-matrisome group in cohort GSE62254. STAD, The Cancer Genome Atlas stomach adenocarcinoma.

In certain cancers, high-matrisome phenotypes are associated with a poor prognosis [23]. We probed the molecular mechanism by which high-matrisome types in gastric cancer are correlated with a poor prognosis [6]. In four cohorts, including our cohort, the high-matrisome group showed poor prognosis (Y497: *p* < 0.0001, STAD: *p* = 0.0053, GSE15459: *p* = 0.035, and GSE62254: *p* = 0.015) (Figure 3a).

We compared the American Joint Committee on Cancer tumor stages by classifying them into high- and low-matrisome groups. In all four cohorts, stage 4 was highly enriched in the high-matrisome group (Figure 3b). We analyzed biological pathways enriched in high-matrisome groups in four cohorts. Notably, elevated gene expression in 22q11.2 copy number variation, ribosome biogenesis, circadian rhythm in Y497 (Table S1), blood vessel development, degradation of the extracellular matrix, molecules associated with elastic fibers, response to growth factor in STAD of TCGA (Table S2), muscle structure development, cell morphogenesis in GSE15459 (Table S3), cytokine signaling in the immune system, and regulation of defense response in GSE62254 (Table S4) were enriched in the high-matrisome group (Figure 3c). We used iRegulon [24] to find transcription factors related to each biological pathway enriched in the high-matrisome group. Transcription factor analysis identified the high expression of the following transcription factors: POLR2A, ETS1 [25], and GABPA (Figure 3d) in Y497; EP300, NFIC, and TCF12 (Figure 3e) in STAD; NFIC, TCF12, and GTF2F1 (Figure 3f) in GSE15459; and IRF1, STAT1, and STAT2 in GSE62254 (Figure 3g).

### 2.4. Identification of Therapeutic Targets in the High-Matrisome Type of Gastric Cancer

**Figure 4:**
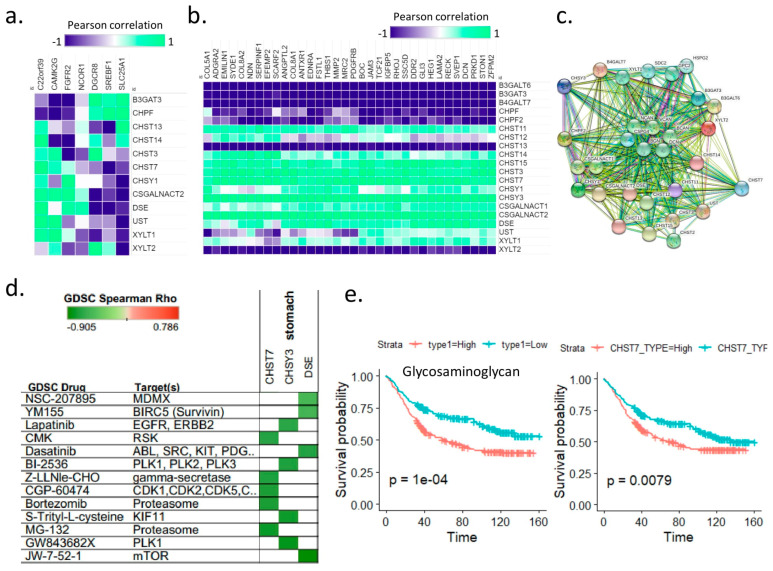
Therapeutic targets in the high-matrisome group in gastric cancer. (**a**) Heat map of Pearson correlation between high expression of glycosaminoglycan biosynthesis-chondroitin sulfate genes and that of 22q11.2 copy number variation pathway genes in cohort Y497. (**b**) Heat map of Pearson correlation between high expression of glycosaminoglycan biosynthesis-chondroitin sulfate genes and that of angiogenesis development genes in cohort STAD. (**c**) Network of protein–protein interaction in glycosaminoglycan biosynthesis-chondroitin sulfate in cohort Y497. (**d**) Predicted drugs from Genomics of Drug Sensitivity in Cancer database for *CHST7*, *CHSY3*, and *DSE*. (**e**) Kaplan–Meier plots for glycosaminoglycan biosynthesis-chondroitin sulfate gene signatures (left) and CHST7 expression in Y497. STAD, The Cancer Genome Atlas stomach adenocarcinoma.

The ECM is considered a promising drug target because it can induce cellular and tissue alterations that can prevent the development or progression of cancer [26]. In this study, we confirmed that the high-matrisome types were often EMT cancer, stem-like-phenotype cancer, and telomerase-absent recalcitrant tumor-type ALT-like cancer. Currently, gastric cancer showing a stem-like phenotype typically shows a poor response to standard treatments and immunotherapy, and effective drugs are largely unavailable; therefore, drug repositioning is necessary. We aimed to identify a drug that can target high-matrisome-type tumors. First, we focused on the glycosaminoglycan biosynthesis-chondroitin sulfate metabolic pathway for candidate drug targets. Pearson correlation analysis was performed for the genes included in the glycosaminoglycan biosynthesis-chondroitin sulfate metabolic pathway and seven genes enriched in the high-matrisome group in cohort Y497; it was confirmed that an increased level of the *C22orf39* gene was positively correlated with that of the glycosaminoglycan biosynthesis gene (Figure 4a).

We also identified angiogenesis and glycosaminoglycan biosynthesis genes that were positively and negatively correlated with the high-matrisome type, respectively (Figure 4b). ECM plays a crucial role in the angiogenic stimulus [27]. The ECM influences the interaction of several growth factors and cytokines [28]. We confirmed a poor prognosis associated with high expression of three genes (*CHST*, *CHSY3*, and *DSE*) included in the glycosaminoglycan biosynthesis-chondroitin sulfate metabolic pathway in the STAD cohort. Protein–protein interaction analysis [29] was performed on these three drug target candidate genes, and the final target selected was *CHST7* (Figure 4c). We found that JW-7-52-1, GW843682, and MG-132 may be effective as drugs for targeting *CHST*, *CHSY3*, and *DSE,* respectively, based on the Genomics of Drug Sensitivity in Cancer (GDSC) database [30]. High expression of *CHST7* was associated with a poor prognosis (*p* = 0.0079) and, thus, its protein may be a promising drug target for treating high-matrisome cancer types (Figure 4e). Our previous study on pan-cancer analysis [9] showed that, when integrated energy metabolism levels are high in a high EMT state, the *CHST14* gene is significantly overexpressed. The high expression of the glycosaminoglycan biosynthesis-chondroitin sulfate metabolic pathway genes was associated with poor prognosis (*p* = 1.0 × 10^−4^) (Figure 4e). Sulfotransferase activity, including that of CHST7, plays a key role in the transformation to an aggressive tumor type in cancer [31].

## 3. Discussion

The core matrisome is composed of genes encoding three components, namely ECM glycoproteins, collagens, and proteoglycans [32]; the ECM regulates cancer progression, metastasis, and development in the tumor microenvironment [33]. In addition, an association between resistance to immunotherapy and the matrisome has been reported recently [23]. However, the involvement of the matrisome in gastric cancer has not been investigated extensively. In this study, we aimed to discover the molecular mechanism by which the matrisome forms the stem-like molecular subtype of gastric cancer and to identify drugs that can inhibit it. The stem-like type shows drug resistance and characteristics of cancer stem cells, and EMT cancer and ALT-like cancer are aggressive tumor types. We found that the high-matrisome type was frequent in stem-like phenotype cancer and was associated with the elevated expression of genes for the ECM-mediated regulation of cell metabolism and stem-cell-like behavior in the tumor microenvironment.

We showed that the high-matrisome type was correlated with high gene expression of five metabolic pathways (glycosaminoglycan biosynthesis-chondroitin sulfate, glycosphingolipid biosynthesis-ganglio series, glycosphingolipid biosynthesis-globo and isoglobo series, glycosaminoglycan biosynthesis-heparan sulfate, and arachidonic acid metabolism). Metabolic reprograming affects the functions of several cells in the vicinity, such as T cells and macrophages [34]. We found that high-matrisome-type gastric cancer was associated with high expression of genes in glycan metabolism, and the correlation between fibroblasts and HSC was high among certain cell types. In addition, the ECM plays a role in regulating cancer hallmarks and it can shift the tumor microenvironment toward contributing to cancer development by inducing EMT and angiogenesis. In general, the high-matrisome type was associated with poor prognosis in gastric cancer, and the prognosis was poor even when glycan metabolism was high. In particular, the high-matrisome samples showed high expression levels of integrated energy genes, such as glucagon-like peptide-1 [35], which regulates the insulin secretion and glucagon signaling pathways. The expression of insulin growth factor [36] is associated with a poor prognosis in gastric cancer and drug-resistant EMT cancer [37], and is also associated with hormonal and metabolic reprograming. We found that sulfotransferase activity in the glycan pathway was associated with poor prognosis; however, in our previous pan-cancer analysis, *CHST14* was shown to be highly expressed in high-energy-metabolism TCGA gastric cancers with high EMT [9]. We confirmed that EMT and energy metabolism were high in gastric cancer with a poor prognosis, which was in agreement with our previous study. We further confirmed that the high-matrisome samples had high glycan metabolic activity. These high-matrisome tumors express factors affecting sulfotransferase activity, thereby contributing to the stem-like phenotype. Although data on the energy source of the stem-like phenotype of gastric cancer are limited, we showed that glycan metabolism acts on the tumor microenvironment as a risk factor more than any other metabolic reprograming indicator.

High glycosaminoglycan biosynthesis-chondroitin sulfate metabolic reprograming was found in high-matrisome samples. High expression of immune checkpoint target genes was found to be correlated with that of glycosaminoglycan biosynthesis-chondroitin sulfate genes, especially the *CHST7* gene. This metabolic reprograming environment inhibits the function of immune cells and is involved in the immune anticancer drug response. Immune cells’ functions and activities are vital in cancer immunotherapy. Through metabolic competition, tumor cells and surrounding stromal cells influence immune cell function and activity in the TME [38].

In summary, this study evaluated the matrisome to identify drugs for the treatment of the stem-like phenotype of gastric cancer. Our findings represent one of the first studies on the matrisome in gastric cancer, and studying the high-matrisome types can serve as an approach for evaluating therapeutic targets against the stem-like phenotype.

## 4. Methods

### 4.1. Data Set Preparation and Matrisome Activity Analysis

We used four cohorts containing gastric cancer transcriptomic data (GSE 15459, GSE62254, TCGA stomach adenocarcinoma, and our cohort Y497). STAD data were obtained from Broad GDAC Firehose (https://gdac.broadinstitute.org/, accessed on 1 August 2021). In the preprocessing step for mRNA expression, genes showing an RNA-seq by an expectation–maximization value of <1 in >50% of the samples were removed. Log2-transformed RNA-seq data were used. We used the GSVA algorithm (R package) to generate a matrisome score using matrisome-related genes. Samples above the mean in each cohort were defined as high-matrisome samples, and samples below the mean were identified as low-matrisome samples.

### 4.2. Metabolic Signature Gene and Cancer Hallmark Analysis

All statistical tests were performed using R version 4.0.1. In total, seven metabolic signatures [39] and 83 KEGG metabolic pathway genes (https://www.genome.jp/kegg/pathway.html accessed on 1 June 2021) were used. To identify statistically significant samples using the GSVA R Package [40], single-sample enrichment was performed by performing >100,000 runs. In total, data on 50 cancer hallmark genes were downloaded from MSigDB (http://software.broadinstitute.org/gsea/msigdb, accessed on 1 August 2021).

### 4.3. Gene Ontology and Transcription Factor Analysis

We used METASCAPE [41] to analyze the canonical biological pathway for gene ontology analysis. First, the R package “limma” was used for differential gene expression analysis of transcriptome data, and the FDR < 0.01 gene was used when the fold-change value of the high-matrisome group was positive.

### 4.4. Survival and Drug Prediction Analysis

We used GEPIA2 for the survival analysis of STAD data (http://gepia2.cancer-pku.cn/#survival accessed on 1 March 2021). The survival R package was used for the Kaplan–Meier survival plot. Drug prediction was performed using GDSC [30].

## 5. Conclusions

The ECM is critical for cancer progression, as it influences tumor metastasis and regulates the tumor microenvironment. In high-matrisome gastric cancers, the ECM may contribute to the formation of aggressive tumors, including EMT, stem-like cancer, and ALT-like cancer types. Our study suggests that, if the high-matrisome type is studied further, drugs may be identified for treating stem-like cancer, a recalcitrant tumor of gastric cancer, and may provide new insights into treating gastric cancer using precision medicine.

## Data Availability

The data presented in this study are available in this article and supplementary material.

## Supplementary Materials

The following are available online at https://www.mdpi.com/article/10.3390/cancers14061438/s1, Table S1: Enriched biological pathway of high matrisome samples in Y497, Table S2: Enriched biological pathway of high matrisome samples in TCGA STAD, Table S3: Enriched biological pathway of high matrisome samples in GSE15459, Table S4: Enriched biological pathway of high matrisome samples in GSE62254.
